# Prediction of Potential miRNA–Disease Associations Through a Novel Unsupervised Deep Learning Framework with Variational Autoencoder

**DOI:** 10.3390/cells8091040

**Published:** 2019-09-06

**Authors:** Li Zhang, Xing Chen, Jun Yin

**Affiliations:** School of Information and Control Engineering, China University of Mining and Technology, Xuzhou 221116, China (L.Z.) (J.Y.)

**Keywords:** miRNA, disease, association prediction, variational autoencoder, generative model

## Abstract

The important role of microRNAs (miRNAs) in the formation, development, diagnosis, and treatment of diseases has attracted much attention among researchers recently. In this study, we present an unsupervised deep learning model of the variational autoencoder for MiRNA–disease association prediction (VAEMDA). Through combining the integrated miRNA similarity and the integrated disease similarity with known miRNA–disease associations, respectively, we constructed two spliced matrices. These matrices were applied to train the variational autoencoder (VAE), respectively. The final predicted association scores between miRNAs and diseases were obtained by integrating the scores from the two trained VAE models. Unlike previous models, VAEMDA can avoid noise introduced by the random selection of negative samples and reveal associations between miRNAs and diseases from the perspective of data distribution. Compared with previous methods, VAEMDA obtained higher area under the receiver operating characteristics curves (AUCs) of 0.9118, 0.8652, and 0.9091 ± 0.0065 in global leave-one-out cross validation (LOOCV), local LOOCV, and five-fold cross validation, respectively. Further, the AUCs of VAEMDA were 0.8250 and 0.8237 in global leave-one-disease-out cross validation (LODOCV), and local LODOCV, respectively. In three different types of case studies on three important diseases, the results showed that most of the top 50 potentially associated miRNAs were verified by databases and the literature.

## 1. Introduction

MicroRNAs (miRNAs), which consist of about 22 nucleotides, are a class of important single-stranded non-coding RNA molecule [1]. Normally, they participate in the regulation of post-transcriptional gene expression through cleaving or translationally repressing target messenger RNAs (mRNAs) [2]. To date, numerous studies have shown that miRNAs influence various biological processes, including cell proliferation, development, differentiation, death, apoptosis, metabolism, aging, signal transduction, and viral infection [3,4,5,6,7]. Therefore, miRNAs have attracted increasing attention, especially with respect to the associations between miRNAs and human diseases. Moreover, some associations between miRNAs and human diseases have been confirmed [8,9,10,11]. For example, in neurological diseases, miR-34a, miR-141 and miR-9 can contribute to Parkinson’s disease-related pathogenic processes by affecting the expression of BCL2, BDNF, and SIRT1 [12]. Further, it was demonstrated that the loss of motor neuron-specific miR-218 might cause systemic neuromuscular failure [13]. Biological experimental verification also showed that both miR-372 and miR-373 act as potential novel oncogenes, participating in the development of human testicular germ cell tumors by numbing the p53 pathway [14]. Another example of miRNA–disease association is that the loss of the Wilms tumor gene on the X chromosome, which is mediated by miR-20a/miR-106a, can regulate colorectal cancer progression and metastasis [15].

There is no doubt that identifying miRNA–disease associations not only enhances the understanding of molecular mechanisms and the pathogenesis of diseases, but also benefits clinical diagnosis and treatment. As is known, carrying out experiments is a scientific and effective method to discover miRNA–disease associations [16,17,18]. Nevertheless, this traditional method has the disadvantages of a long cycle and high cost, such that it cannot meet the needs of researchers to efficiently discover miRNA–disease associations. To be more specific, experimental methods such as the anchored polymerase chain reaction, competitive polymerase chain reaction, and reverse transcription polymerase chain reaction have been widely used by researchers, yet they have complex experimental steps and need a large number of chemical reagents [19,20,21,22]. Without doubt, these experiments are time-consuming and expensive. With the accumulation of biological data and the improvement of computing ability, utilizing computational models to predict disease-related miRNAs has begun to develop [23]. As a supplement to experimental methods, computational approaches can provide reasonable candidate disease-related miRNAs for future biological experiments.

According to the statistics, computational models for predicting miRNA–disease associations have emerged in recent years. Some of these models based on different principles performed well in predicting miRNA–disease associations [24]. As follows, the first three models introduced are based on random walk analysis. Unlike previous local network similarity measures, Chen et al. [25] developed a computational model of random walk with restart for MiRNA–disease association (RWRMDA). They took full advantage of global network similarity measures and performed random walk with restart on the miRNA functional similarity network in order to predict potential associations between miRNAs and diseases. Afterwards, Shi et al. [26] developed a method to identify human miRNA–disease associations on basis of functional links between miRNA targets and disease genes in a protein–protein interaction (PPI) network. They performed a random walk with the restart algorithm in the PPI network to calculate the association scores between miRNAs and diseases. Last but not least, Xuan et al. [27] released another random walk-based model named MIRNAs associated with disease prediction (MIDP). Specifically, according to the known associations between miRNAs and the investigated disease, they first classified all nodes of a miRNA functional similarity network into labeled nodes and unlabeled nodes. Then, two transition matrices were constructed for labeled nodes and unlabeled nodes, respectively. Further, the labeled nodes were assigned higher transition weights than the unlabeled nodes. After performing the random walk on the miRNA functional similarity network, the association scores between each unlabeled node and the investigated disease could be obtained. Through constructing a miRNA–disease bilayer network, they proposed an extension method named MIDPE based on MIDP. Moreover, MIDPE can be utilized to select candidate miRNAs for diseases without any known associated miRNAs.

Later, Xuan et al. [28] devised a computational model named human disease-related MiRNA prediction (HDMP) that is one of the typical representatives to use the scoring function. Given an investigated disease, *k*, most similar neighbors of each unlabeled miRNA were selected on the basis of miRNA functional similarity. In the process of computing sub-scores between *k*’s most similar neighbors and the given disease, the neighbors that belong to the same cluster or family with the unlabeled miRNA would be assigned higher weights. Finally, by summing up the sub-scores, the final association score between the unlabeled miRNA and the investigated disease could be obtained. Because the calculation of sub-scores relied on known associations between the investigated disease and miRNA’s *k* neighbors, HDMP cannot be applied to a new disease without any known associated miRNAs. Then, Mork et al. [29] provided a protein-driven approach called miRNA–protein–disease (miRPD) to predict potential associations between miRNAs and diseases. Using protein as a medium, they designed a scoring scheme by combining the miRNA–protein association scores and protein–disease association scores. Furthermore, Chen et al. [30] put forward a method of within and between score for MiRNA–disease association prediction (WBSMDA). In detail, for an unknown miRNA–disease pair, they calculated the within score and between score, respectively. From the perspective of miRNA, the highest similarity score between the investigated miRNA and the known related miRNAs of the investigated disease was defined as the within score. Further, the highest similarity score among the similarity scores between the investigated miRNA and the unrelated-miRNAs of the investigated disease was defined as Between-Score. Similarly, the within score and between score could also be obtained in view of the disease. Then, based on the within score and between score, the final association score of the unknown miRNAdisease pair could be obtained. Moreover, Pasquier and Gardes [31] released a model called MiRAI to predict potential miRNA–disease associations. In the model, through incorporating a miRNA–disease association matrix, miRNA–neighbor association matrix, miRNA–target association matrix, miRNA–word association matrix, and miRNA–family association matrix, they obtained a spliced matrix with the aforementioned information. Then, they reduced the dimension of this matrix with singular value decomposition to obtain miRNA vectors and disease vectors. Finally, the association score of a miRNA–disease pair could be obtained by calculating the cosine distance between a miRNA vector and a disease vector.

Here are also some network algorithm-based models to predict potential associations between miRNAs and diseases. Gu et al. [32] presented the network consistency projection for miRNA–disease associations (NCPMDA) method to predict potentially related miRNAs for diseases. They first constructed a miRNA similarity network and a disease similarity network. After that, on the basis of network consistency, the two similarity networks were projected to the miRNA–disease association network, respectively. At last, the final prediction scores were given via combing and normalizing the miRNA space projection scores and the disease space projection scores. Yu et al. [33] also came up with a complex network model named MaxFlow to enable large-scale prediction of miRNA–disease associations. Firstly, they built the heterogeneous miRNA–disease association network by integrating miRNA–disease associations, miRNA family, and cluster information. Next, through combining the miRNA functional similarity network, the disease semantic and phenotypic similarity network and the heterogeneous miRNA–disease association network, the microRNAome-phenome network graph was constructed. For an investigated disease, through introducing a source node and a sink node to this graph, the maximum information flow from the source over all links to the sink were calculated using the push-relabel maximum flow algorithm. The flow quantity leaving a miRNA node was used as the association score between the miRNA and the investigated disease. Afterwards, bipartite network projection for MiRNA–disease association prediction (BNPMDA) was proposed by Chen et al. [34]. In the beginning, Chen et al. constructed bias ratings for miRNAs and diseases by using agglomerative hierarchical clustering. Then, in view of the bias ratings, they allocated transfer weights to resource allocation links between miRNAs and diseases, and executed the bipartite network recommendation algorithm to achieve association scores between miRNAs and diseases. What’s more, taking full advantage of the sparse learning method to eliminate the noise of the miRNA–disease adjacency matrix before the heterogeneous graph inference method, Chen et al. [35] proposed matrix decomposition and heterogeneous graph inference for miRNA–disease association prediction (MDHGI).

Additionally, computational models for miRNA–disease association prediction using machine learning methods have also begun to appear. Firstly, Chen et al. [36] designed a semi-supervised classifier model of regularized least squares for MiRNA–disease association prediction (RLSMDA). Under the framework of regularized least squares, they constructed two optimal classification functions in miRNA space and disease space, respectively. Afterwards, through taking a simple weighted average operation, two optimal classifiers in different spaces were combined to infer miRNA–disease associations. Analogously, Chen et al. [37] developed another semi-supervised model of inductive matrix completion for MiRNA–disease association prediction (IMCMDA). They first constructed the objective function to find an approximation of the known miRNA–disease association matrix. Then, by employing gradient descent, they calculated the optimal solution of the objective function to obtain the low dimensional representation of miRNAs and diseases. Last, by means of matrix multiplication, the two low dimensional representation matrices were directly incorporated by the similarity matrix of miRNAs and diseases to obtain final relevance scores of miRNA–disease pairs.

Supervised machine learning models require reliable negative samples compared to the semi-supervised machine learning methods above. Recently, Chen et al. [38] developed a supervised machining learning model named ranking-based KNN for miRNA–disease association prediction (RKNNMDA). Initially, they utilized the *k*-nearest neighbors (KNN) algorithm to select *k*-nearest-neighbors for miRNAs and diseases. Secondly, based on the support vector machine (SVM) ranking model, they reranked the *k*-nearest-neighbors respectively for miRNAs and diseases. Then, the weighted voting method was applied to calculate the association scores between miRNAs and diseases. Then, Chen et al. [39] released random forest for MiRNA–disease association prediction (RFMDA), which was a supervised classifier model. In the model, negative samples were randomly selected from unlabeled miRNA–disease pairs and positive samples included all known miRNA–disease associations. Next, based on integrated miRNA similarity and integrated disease similarity, they constructed feature vectors for negative samples and positive samples, respectively. Then, a filter-based method was implemented to reduce the dimension of feature vectors. Finally, the random forest was trained to infer potential relationships of unlabeled miRNA–disease pairs. Further, Wang et al. [40] developed another supervised model of negative samples extraction based MiRNA–disease association prediction (NSEMDA). Each sample was represented by a feature vector on the basis of integrated miRNA similarity and integrated disease similarity. By means of calculating the specificity score, they chose robust features for each sample. Using the spy classifier and Rocchio classifier, they selected reliable negative samples, reliable positive samples, and ambiguous samples from an unlabeled sample set. After constructing the support vector machine-similarity weight (SVM-SW) model, they trained the model with a training sample set that included positive samples, negative samples, and ambiguous samples with similarity weight. Finally, SVM-SW was used to infer potential associations between miRNAs and diseases.

Although some computational models for miRNA–disease association prediction have been developed, most of them still need to be improved in order to serve the purposes of biological experiment and clinical application. On the one hand, some computational models cannot predict miRNAs potentially associated with new diseases. On the other hand, supervised machine learning methods require reliable positive and negative samples, but there are no real negative samples in the miRNA–disease association prediction. Selecting negative samples from unknown miRNA–disease pairs may introduce errors that reduce the predictive effect. In order to overcome these problems, we proposed the variational autoencoder for MiRNA–disease association prediction (VAEMDA), which is an unsupervised deep learning model to predict the associations between diseases and miRNAs. According to statistics, VAE has been well applied in different fields. Through combing high-throughput cell line assays of drug-induced transcriptomic perturbation effects, the drug response variational autoencoder (Dr.VAE) has been developed to improve drug response prediction [41]. Rashid et al. have well applied VAE to unmask tumor heterogeneity from single cell genomic data [42]. Further, Tezcan et al. have succeeded in reconstructing magnetic resonance (MR) images from under-sampled measurements with VAE [43]. Xu et al. also developed a novel model of the semi-supervised sequential variational autoencoder (SSVAE) to improve the accuracy of text classification [44]. In our study, first of all, we constructed two spliced matrices by combining the integrated miRNA similarity and the integrated disease similarity to the known miRNA–disease associations, respectively. Then, we used the two spliced matrices as the input of the variational autoencoder (VAE). The VAE learned latent representations and distributions of the input data, and then sampled from the learned distribution to reconstruct the original input data [45]. After completing the training process, we can apply the optimal VAE model to score for unknown miRNA–disease pairs. In this article, three kinds of cross validation scheme and three types of case studies were used to evaluate our model. In global and local leave-one-out cross validation (LOOCV), VAEMDA obtained AUCs of 0.9118 and 0.8652, respectively. By carrying out 100 times five-fold cross validation, VAEMDA obtained an average AUC of 0.9091 and standard deviation of 0.0065. In global and local leave-one disease-out cross validation (LODOCV), VAEMDA obtained AUCs of 0.8250 and 0.8237. In case studies, VAEMDA was applied to three high incidence cancers (i.e., esophageal neoplasm (EN), hepatocellular carcinoma (HC) and breast neoplasm (BN)). The results showed that 45, 50, and 48 of the top 50 potentially associated miRNAs of the three investigated diseases were verified by databases and experimental literature, respectively. In conclusion, VAEMDA can become an effective tool for predicting miRNA–disease associations and could assist in biological experiments and clinical related studies.

## 2. Materials and Methods

### 2.1. Data Preparation

This section is divided into a total of six parts to introduce the data used in this article including human miRNA–disease associations, disease semantic similarity 1, disease semantic similarity 2, miRNA functional similarity, Gaussian interaction profile kernel similarity for miRNAs and diseases and integrated similarity for miRNAs and diseases.

Human miRNA–disease associations: We downloaded them from the HMDD v2.0 database [46] involving 495 miRNAs, 383 diseases, and 5430 known miRNA–disease associations verified by biological experiments. We used nd and nm to denote the number of diseases and miRNAs, respectively. To clearly illustrate the relationship between miRNAs and diseases, an adjacency matrix A with a size of nm rows and nd columns was constructed in which the element A(m(i),d(j)) is equal to 1 if miRNA m(i) and disease d(j) were verified to be related, otherwise 0.

Disease semantic similarity 1: We used MeSH descriptors from the National Library of Medicine (http://www.nlm.nih.gov/) to achieve directed acyclic graphs (DAGs) of diseases. Based on the assumption that the larger parts the DAGs of two diseases share in common, the larger semantic similarity value between the two diseases, we made full use of the method in previous study [28] to calculate the disease semantic similarity between different diseases. Then, we can get a nd×nd disease semantic similarity matrix SS1.

Disease semantic similarity 2: Considering that different disease terms in the same layer of DAG may appear in the different numbers of disease DAGs, we adopted another method in previous study [28] to calculate the disease semantic similarity between different diseases. As a result, we obtained a disease semantic similarity matrix SS2.

MiRNA functional similarity: In our study, miRNA functional similarity scores were calculated by the method in previous study [47]. We downloaded the similarity scores from the website of http://www.cuilab.cn/files/images/cuilab/misim.zip and constructed a miRNA functional similarity matrix FS.

Gaussian interaction profile kernel similarity for miRNAs and diseases: Under the assumption that diseases associated with the same miRNA are more likely to be similar, and vice versa, we used adjacency matrix A mentioned above to calculate Gaussian interaction profile kernel similarity for diseases according to the previous method [48]. KD was used to represent the nd×nd Gaussian interaction profile kernel similarity matrix for diseases. Then, we used the same method to calculate Gaussian interaction profile kernel similarity for miRNAs. KM represented the nm×nm Gaussian interaction profile kernel similarity matrix for miRNAs.

Integrated similarity for miRNAs and diseases: In order to obtain the nd×nd integrated disease similarity matrix SD, we combined disease semantic similarity 1, disease semantic similarity 2 and Gaussian interaction profile kernel similarity of diseases according to the method in previous study [30]. Similarly, we integrated miRNA functional similarity and Gaussian interaction profile kernel similarity of miRNAs to construct the nm×nm integrated miRNA similarity matrix SM.

### 2.2. VAEMDA

**Figure 1:**
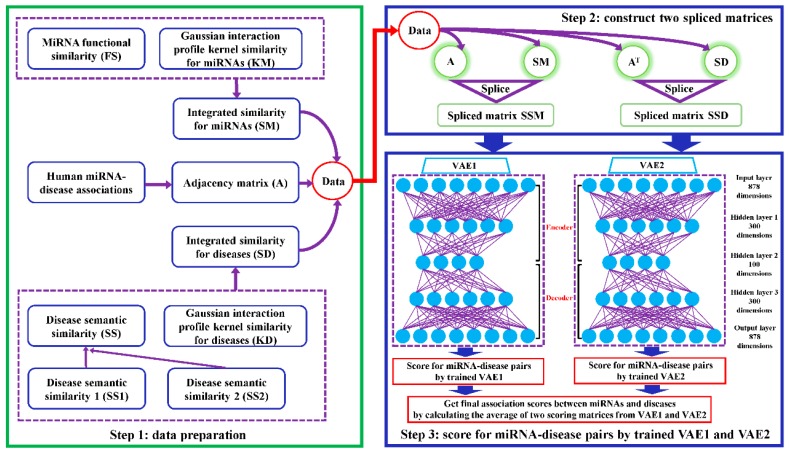
Flowchart of potential miRNA–disease association prediction based on the computational model of VAEMDA: (1) Data preparation, where integrated similarity for miRNAs/diseases (SM/SD) were calculated and the adjacency matrix A representing human miRNA–disease associations was constructed. (2) Construct two spliced matrices, where adjacency matrix A and integrated similarity matrix SM for miRNAs were spliced into matrix SSM. At the same time, adjacency matrix $A^{T}$ and integrated similarity matrix SD for diseases were spliced into matrix SSD. (3) Score for miRNA–disease pairs by trained VAE1 and trained VAE2, where spliced matrix SSM and spliced matrix SSD were applied to train VAE1 and VAE2, respectively. We calculated the average of two scoring matrices to obtain final association scores between miRNAs and diseases.

In this paper, we developed a computational model named VAEMDA to predict potential miRNA–disease associations (motivated by the study of Titus et al. [45]). The implementation process of VAEMDA can be divided into three steps. The flowchart of VAEMDA is shown in Figure 1.

Firstly, based on data collection and similarity calculation, we can get the adjacency matrix A, integrated similarity matrix SM and integrated similarity matrix SD.

Secondly, we utilized matrix A, SM, and SD to construct two spliced matrices, which were treated as the training data of VAEMDA. Specifically, matrix A and matrix SM were spliced into the matrix SSM with a size of nm rows and nd+nm columns, where the first nd columns belong to matrix A and the last nm columns come from matrix SM. We constructed another nd×(nm+nd) matrix SSD by splicing matrix $A^{T}$ and matrix SD, where the first nm columns come from matrix $A^{T}$ and the last nd columns belong to matrix SD. Moreover, the purpose of constructing spliced matrix SSM and SSD is to combine the integrated miRNA similarity matrix and the integrated disease similarity matrix with the adjacency matrix of miRNA–disease association network, respectively.

Thirdly, we utilized the VAE model to learn potential associations between miRNAs and diseases. VAEMDA contains two VAE models named VAE1 and VAE2 respectively, where we used spliced matrix SSM to train VAE1 and used spliced matrix SSD to train VAE2. Both VAE1 and VAE2 consist of an Adam optimizer [49], rectified linear units [50], batch normalization in the encoding stage, and sigmoid activations in the decoding stage. VAE1 and VAE2 also have the same loss function, consisting of two parts: one is the data reconstruction error, which can measure the error between the original data and the reconstructed data, and the other is the Kullback–Leibler divergence, which can measure the difference between the distribution of potential variables and the standard normal distribution. Both VAE1 and VAE2 were built in Keras (Version 2.0.6) [45] with a Tensorflow backend (Version 1.2.1) [51]. According to the optimal parameters in previous study [45], we trained VAE1 and VAE2 with the following values: batch size = 20, learning rate = 0.001, standard deviation = 1, epochs = 50, train/validation = 9/1. As described in Step 3 of Figure 1, based on the characteristics of our training samples, we determined the number of hidden layers (i.e., three hidden layers) and the number of neurons in each hidden layer (i.e., 300 neurons in hidden layer 1100 neurons in hidden layer 2300 neurons in hidden layer 3). In addition to the three hidden layers, the VAE in our model also contains the input layer (878 dimensions) and the output layer (878 dimensions). The process of transforming from the input layer to the hidden layer 2 is defined as the encoder (i.e., the process from input layer to hidden layer 1 and another process from hidden layer 1 to hidden layer 2), in which the VAE in our model can learn the distribution characteristics of training data. The process of transforming from the hidden layer 2 to the output layer is defined as the decoder (i.e., the process from hidden layer 2 to hidden layer 3 and another process form hidden layer 3 to output layer), in which the VAE in our model can regenerate the input data and supplement missing values in the input data. We utilized SSM and SSD to train VAE1 and VAE2, respectively until the loss function of the two VAE models converged. After completing the training, we can score for unknown miRNA–disease pairs with VAE1 and VAE2. Taking full advantage of miRNA integrated similarity and disease integrated similarity, we calculated the average of two scoring matrices to obtain the final association scores between miRNAs and diseases.

## 3. Results

### 3.1. Performance Evaluation

We adopted LOOCV and five-fold cross validation to evaluate the performance of VAEMDA, based on HMDD v2.0 [46] involving 5430 known miRNA–disease associations between 383 diseases and 495 miRNAs. In our research, LOOCV contains global and local LOOCV. In global LOOCV, we took each sample among 5430 known miRNA–disease associations in turn as the test sample. Simultaneously, the remaining 5429 known miRNA–disease associations and all unknown miRNA–disease disease pairs were regarded as training samples. Then, the trained VAEMDA was applied to score for all unknown miRNA–disease pairs and the test sample. Through comparing the scores of each test sample with all unknown miRNA–disease pairs, we would get the rank of the test sample. Yet, in local LOOCV, the rank of the test sample can be achieved by comparing the scores of the test sample with unknown miRNA–disease pairs involving the investigated disease.

In five-fold cross validation, all known miRNA–disease associations were randomly divided into five subsets with equal size. Then, each subset containing 1086 known miRNA–disease associations was selected as the test sample in turn. Besides, the other four subsets and all unknown miRNA–disease pairs were considered as training samples. In the same way as LOOCV, the trained VAEMDA was used to score for test samples and all unknown miRNA–disease pairs. Through comparing the scores of each test sample with all unknown miRNA–disease pairs, the rank of each test sample could be achieved. Due to random division of samples, we performed five-fold cross validation 100 times.

In LOOCV and five-fold cross validation, with each change of the adjacency matrix between miRNAs and diseases, we would recalculate the Gaussian interaction kernel similarity every time. The rank exceeding the given threshold would indicate a successful prediction made by the model and vice versa. Then, we drew a receiver operating characteristics (ROC) curve at different thresholds with the true positive rate (TPR) as the X-axis and the false positive rate (FPR) as Y-axis. Moreover, we evaluated the predictive performance of computational model through calculating the area under the ROC curve (AUC). An AUC of 1 means a perfect performance whereas an AUC of 0.5 implies a random performance.

**Figure 2:**
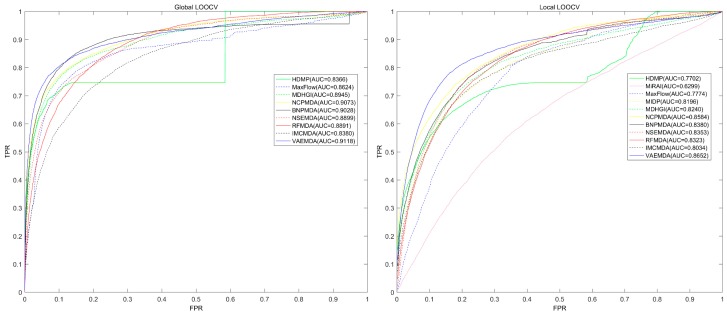
Performance comparison between VAEMDA and ten previous disease-miRNA association prediction models (NCPMDA, BNPMDA, MDHGI, NSEMDA, RFMDA, MaxFlow, IMCMDA, HDMP, MiRAI and MIDP) in terms of ROC curves and AUCs based on global and local LOOCV. As a result, VAEMDA outperformed other models by achieving an AUC of 0.9118 in global LOOCV and an AUC of 0.8652 in local LOOCV.

As shown in Figure 2, in global LOOCV, the AUC of VAEMDA (0.9118) was higher than HDMP (0.8366), MaxFlow (0.8624), MDHGI (0.8945), NCPMDA (0.9073), BNPMDA (0.9028), NSEMDA (0.8899), RFMDA (0.8891) and IMCMDA (0.8380). In local LOOCV, our model also got the largest AUC (0.8652) compared to HDMP (0.7702), MiRAI (0.6299), MaxFlow (0.7774), MIDP (0.8196), MDHGI (0.8240), NCPMDA (0.8584), BNPMDA (0.8380), NSEMDA (0.8353), RFMDA (0.8323) and IMCMDA (0.8034). In 5-fold cross validation, we repeated this procedure for 100 times to achieve a sound estimate of the average prediction accuracy of VAEMDA and obtained an AUC of 0.909 ± 0.0065, surpassing that for NCPMDA (0.8763 ± 0.0008), BNPMDA (0.8980 ± 0.0013), MDHGI (0.8794 ± 0.0021), NSEMDA (0.8878 ± 0.0014), RFMDA (0.8818 ± 0.0014), MaxFlow (0.8579 ± 0.001), IMCMDA (0.8367 ± 0.0005) and HDMP (0.8342 ± 0.0010). Global LOOCV was not applicable to MiRAI, because association scores given by this model were highly positively correlated with the number of known associated miRNAs of the investigated disease. Further, for a disease with more known associated miRNAs, the association scores predicted by MiRAI tended to be higher. These led to the incomparability of association scores predicted by MiRAI between different diseases. In addition, the core of MiRAI was collaborative filtering that suffers from data sparsity problem. The reason why global LOOCV was not applicable to MIDP is that the method was based on random walk which was a local approach and could not simultaneously make predictions for all diseases [52].

Similarly, in global LODOCV and local LODOCV, we divided all known miRNA–disease associations into 383 subsets, each of which contained all known associations between miRNAs and one investigated disease. When global LODOCV was used to evaluate the performance of VAEMDA, we treated all known associations in each subset as the test samples in turn. The rest 382 subsets and all unknown miRNA–disease pairs were considered as training samples. Then, the trained VAEMDA was implemented to score for all unknown miRNA–disease pairs and test samples. Through comparing the scores of each test sample with all unknown miRNA–disease pairs, we would get the rank of each test sample. Unlike global LODOCV, the rank of each test sample would be obtained by means of comparing the scores of each test sample with unknown miRNA–disease pairs involving the investigated disease in local LODOCV. In global and local LODOCV, VAEMDA obtained AUCs of 0.8250 and 0.8237, respectively.

### 3.2. Case Studies

To further validate the performance of our model in predicting new miRNA–disease associations, we conducted three different types of case studies on three important diseases.

In the first type of case study on EN, based on known associations in HMDD v2.0, VAEMDA was implemented to score for candidate miRNAs of EN and the top 50 potentially EN-related miRNAs were selected to make further validation. According to the survey [53], it was estimated that 17,650 new cases of EN would appear and 16,080 persons would die from EN in the United States in 2019. Recently, some researchers have shown that the overexpression of miR-377 inhibits the initiation, growth, and angiogenesis of EN, whereas the silencing of miR-377 has opposite effects [54]. The finding indicates that miR-377 might serve as a promising non-invasive diagnostic and prognostic biomarker in the clinical treatment of EN [54]. Further, it has been observed that SOX4 silences miR-31 to indirectly promote proliferation and invasion of EN cells [55]. The result of the first type of case study showed that 10 out of the top 10, 19 out of the top 20, and 45 out of the top 50 predictions were confirmed by databases (dbDEMC, miR2Disease) and the literature (see Table 1).

In the second type of case study, we want to illustrate the ability of VAEMDA in identifying associated miRNAs for new diseases without any known related miRNAs. Taking HC as the example, we set all the known associations between miRNAs and HC as unknown ones so that HC could be treated as a new disease. Then, VAEMDA was performed to score for all HC-miRNA pairs and the top 50 potentially HC-related miRNAs were selected to make further validation. The American Cancer Society forecasted that the number of deaths caused by HC in the United States would reach 31,780 in 2019 [53]. Globally, the incidence of breast cancer is still rising by 0.2–0.8% every year [56]. In recent years, some medical researchers have found that, compared with normal people, the expression of miR-1296 is reduced in HC patients [57]. Increasing the expression of miR-1296 would inhibit migration, invasion and EMT progress of HC cells [57]. Therefore, it was concluded that the expression level of miR-1296 might serve as a prognostic biomarker in HC [57]. In this type of case study, it was observed that 10 out of the top 10, 20 out of the top 20 and 50 out of the top 50 predicted miRNAs were verified by databases (dbDEMC, miR2Disease and HMDD v2.0) and the literature (see Table 2).

Based on the known associations in HMDD v1.0, we implemented the third type of case study to verify the generalization ability of VAEMDA (i.e., the prediction ability when VAEMDA was applied to different datasets). Here, taking BN as the investigated disease, the result showed that there are 10, 20 and 48 out of the top 10, 20 and 50 predicted BN-related miRNAs confirmed by databases (dbDEMC, miR2Disease and HMDD v2.0) and literatures (see Table 3). Among the top 50 predicted miRNAs, we selected the first-ranked hsa-let-7b and the second-ranked hsa-let-7g to further illustrate the specific process of their associations with diseases. Xu et al. [58] have proven that p62 expression was elevated in BN stem cells, and increasing the expression of has-let-7b in BN stem cells can inhibit the expression of p62. This may provide a new therapeutic approach for BN treatment [58]. Moreover, cancer-associated fibroblasts (CAFs) can promote tumorigenesis, growth, invasion, and metastasis of BN [59]. Through conducting pathway analysis, Zhao et al. [59] found that hsa-let-7g was down-regulated in CAFs, suggesting that hsa-let-7g can induce BN by affecting CAFs.

In conclusion, from the above evaluation results, we can clearly see that VAEMDA shows reliable performance in both cross validation and case studies. With the continuous experimental research on miRNA–disease associations, we believe that more and more potential miRNA–disease associations predicted by our model could be confirmed in the future.

## 4. Discussion

It is important for clinical diagnoses and treatments of diseases to discover disease-associated miRNAs. In this paper, we developed an unsupervised computational model called VAEMDA to predict potential associations between miRNAs and diseases. The implementation process of our model can be divided into three parts. The first part was to construct two spliced matrices. Through respectively combining the integrated miRNA similarity and the integrated disease similarity with the miRNA–disease associations, we constructed two spliced matrices that were regarded as the input of our model. The second part was to build and optimize the model. After constructing the VAE deep learning framework, we utilized the Adam strategy to optimize our model based on training data (i.e., the two spliced matrices). The third part was to use trained VAEMDA to score for candidate miRNA–disease pairs and obtain potential miRNA–disease associations. To evaluate the performance of VAEMDA, we carried out three kinds of cross validation and three types of case studies on EN, HC and BN. The AUCs of VAEMDA were 0.9118 and 0.8652 in global LOOCV and local LOOCV, respectively, which exceeded ten previous models. In 100 times five-fold cross validation, VAEMDA obtained the average AUC of 0.9091 and standard deviation of 0.0065, which reflected the stability of VAEMDA in prediction to some extent. Further, the AUCs of VAEMDA were 0.8250 and 0.8237 in global LODOCV and local LODOCV. In case studies, based on the association scores predicted by VAEMDA, we selected the top 50 potential associated miRNAs for each disease, most of which were confirmed by databases and experimental literatures. Three types of case studies on three different important diseases further illustrated the excellent performance of VAEMDA.

VAEMDA shows more reliable performance over previous methods because of the following four factors. First, the unsupervised model of VAEMDA does not need negative samples. Therefore, VAEMDA can avoid the noise introduced by randomly selecting negative samples. Second, the core of VAEMDA is the deep learning framework named VAE that is a kind of generation models. Generation models have great advantages in dealing with data loss problems. We constructed two spliced matrices as the training data of our model, and a part of the data in each spliced matrix is missing. Therefore, the feature of our training data fully exerts the role of VAE, which makes VAMEDA more reliable. Third, as a data-driven deep learning model, VAEMDA repeatedly adjust a weighted combination of input features until the model identifies the best possible reconstruction of the input data. Further, the reconstruction process of original data in VAEMDA can be treated as a high dimensional interaction space in which the latent dimensions capture the complex associations between miRNAs and diseases. Fourth, in VAE, the process of reconstructing the original data has a confrontational relationship with the process of calculating the Kullback–Leibler loss. In detail, the process of reconstructing original data is continuously to reduce noise, while the process of calculating the Kullback–Leibler loss will continuously generate Gaussian noise. This kind of confrontational relationship actually avoids the over-fitting of VAEMDA during the training process and improves the robustness and generalization ability of VAEMDA.

However, VAEMDA still has a lot of room for improvement. In this paper, the miRNA and disease similarity calculation might not be the perfect method. In the future, we expect more biological information to be added to fine-tune the similarity measure. In addition, in the HMDD v2.0 database, the known miRNA–disease associations account for less than 3% of all miRNA–disease pairs. In the future, as more and more novel miRNA–disease associations are discovered, we believe the predictive accuracy and stability of VAEMDA can be improved. Moreover, although deep learning models have been used in some areas and achieved good results, this kind of model still lacks interpretability due to the nonlinear nature of the model architecture, and VAEMDA is no exception. We are currently working to make VAEMDA more interpretive, accessible, and useful to biologists.

## Figures and Tables

**Table 1:** Prediction of the top 50 potential EN-related miRNAs.

miRNA	Evidence	miRNA	Evidence
hsa-mir-195	dbDEMC	hsa-mir-144	dbDEMC
hsa-mir-221	dbDEMC	hsa-mir-30d	dbDEMC
hsa-mir-146b	dbDEMC	hsa-mir-7	dbDEMC
hsa-mir-125b	dbDEMC	hsa-mir-337	unconfirmed
hsa-mir-200b	dbDEMC	hsa-mir-107	dbDEMC; miR2Disease
hsa-mir-9	dbDEMC	hsa-mir-30c	dbDEMC
hsa-mir-29b	dbDEMC	hsa-mir-378a	unconfirmed
hsa-mir-24	dbDEMC	hsa-mir-513a	unconfirmed
hsa-mir-106b	dbDEMC	hsa-mir-16	dbDEMC
hsa-mir-30a	dbDEMC	hsa-mir-204	26722467
hsa-mir-429	dbDEMC	hsa-mir-367	dbDEMC
hsa-mir-206	dbDEMC	hsa-mir-422a	dbDEMC
hsa-mir-182	dbDEMC	hsa-let-7g	dbDEMC
hsa-mir-103a	unconfirmed	hsa-mir-127	dbDEMC
hsa-let-7e	dbDEMC	hsa-mir-142	dbDEMC
hsa-mir-27b	dbDEMC	hsa-mir-198	dbDEMC
hsa-mir-193b	dbDEMC	hsa-mir-125a	dbDEMC
hsa-mir-224	dbDEMC	hsa-mir-23a	dbDEMC
hsa-mir-10b	dbDEMC	hsa-mir-197	dbDEMC
hsa-mir-1	dbDEMC	hsa-mir-96	dbDEMC
hsa-mir-424	dbDEMC	hsa-mir-20b	dbDEMC
hsa-mir-708	27092874	hsa-mir-133b	dbDEMC
hsa-mir-32	dbDEMC	hsa-mir-191	dbDEMC
hsa-mir-17	dbDEMC	hsa-mir-132	dbDEMC
hsa-mir-222	dbDEMC	hsa-mir-103b	unconfirmed

The first column records top 1–25 related miRNAs. The third column records the top 26–50 related miRNAs. The evidences for the associations were either dbDEMC and miR2Disease or more recent experimental literatures with the corresponding PMIDs.

**Table 2:** Prediction of the top 50 potential HC-related miRNAs.

miRNA	Evidence	miRNA	Evidence
hsa-mir-484	HMDD v2.0	hsa-mir-608	HMDD v2.0
hsa-mir-148a	dbDEMC; miR2Disease; HMDD v2.0	hsa-mir-218	HMDD v2.0
hsa-mir-29b	dbDEMC; HMDD v2.0	hsa-mir-21	miR2Disease; HMDD v2.0
hsa-let-7b	miR2Disease; HMDD v2.0	hsa-mir-490	HMDD v2.0
hsa-mir-181b	dbDEMC; miR2Disease; HMDD v2.0	hsa-mir-301a	HMDD v2.0
hsa-mir-483	HMDD v2.0	hsa-mir-10b	HMDD v2.0
hsa-mir-96	miR2Disease;HMDD v2.0	hsa-mir-638	28529597
hsa-mir-34b	28337312	hsa-mir-221	dbDEMC; miR2Disease; HMDD v2.0
hsa-let-7e	dbDEMC; miR2Disease; HMDD v2.0	hsa-mir-326	HMDD v2.0
hsa-mir-320e	HMDD v2.0	hsa-mir-362	HMDD v2.0
hsa-mir-1271	HMDD v2.0	hsa-mir-26	HMDD v2.0
hsa-mir-30c	miR2Disease; HMDD v2.0	hsa-mir-320b	HMDD v2.0
hsa-mir-26a	dbDEMC; miR2Disease; HMDD v2.0	hsa-mir-320d	HMDD v2.0
hsa-mir-450b	HMDD v2.0	hsa-mir-1202	HMDD v2.0
hsa-mir-629	HMDD v2.0	hsa-mir-519e	HMDD v2.0
hsa-mir-409	HMDD v2.0	hsa-mir-187	HMDD v2.0
hsa-mir-503	HMDD v2.0	hsa-let-7g	miR2Disease; HMDD v2.0
hsa-mir-320c	HMDD v2.0	hsa-mir-92	dbDEMC; HMDD v2.0
hsa-mir-219	miR2Disease; HMDD v2.0	hsa-mir-302b	HMDD v2.0
hsa-mir-181d	dbDEMC; HMDD v2.0	hsa-mir-125a	dbDEMC; miR2Disease; HMDD v2.0
hsa-mir-491	HMDD v2.0	hsa-let-7d	miR2Disease; HMDD v2.0
hsa-let-7a	dbDEMC; miR2Disease; HMDD v2.0	hsa-mir-345	HMDD v2.0
hsa-mir-526a	HMDD v2.0	hsa-mir-527	HMDD v2.0
hsa-mir-450a	HMDD v2.0	hsa-mir-34c	HMDD v2.0
hsa-let-7f	miR2Disease; HMDD v2.0	hsa-let-7c	dbDEMC; miR2Disease; HMDD v2.0

The first column records top 1–25 related miRNAs. The third column records the top 26–50 related miRNAs. The evidences for the associations were dbDEMC, miR2Disease and HMDD v2.0 or more recent experimental literatures with the corresponding PMIDs.

**Table 3:** Prediction of the top 50 potential BN-related miRNAs.

miRNA	Evidence	miRNA	Evidence
hsa-let-7b	dbDEMC; HMDD v2.0	hsa-mir-126	dbDEMC; miR2Disease; HMDD v2.0
hsa-let-7g	dbDEMC; HMDD v2.0	hsa-mir-135a	dbDEMC; HMDD v2.0
hsa-mir-92b	dbDEMC	hsa-mir-128b	miR2Disease
hsa-mir-16	dbDEMC; HMDD v2.0	hsa-mir-24	dbDEMC; HMDD v2.0
hsa-let-7i	dbDEMC; miR2Disease; HMDD v2.0	hsa-mir-191	dbDEMC; miR2Disease; HMDD v2.0
hsa-let-7e	dbDEMC; HMDD v2.0	hsa-mir-182	dbDEMC; miR2Disease; HMDD v2.0
hsa-mir-223	dbDEMC; HMDD v2.0	hsa-mir-27a	dbDEMC; miR2Disease; HMDD v2.0
hsa-mir-99a	dbDEMC	hsa-mir-26a	dbDEMC; miR2Disease; HMDD v2.0
hsa-mir-100	dbDEMC; HMDD v2.0	hsa-mir-195	dbDEMC; miR2Disease; HMDD v2.0
hsa-mir-92a	HMDD v2.0	hsa-mir-150	dbDEMC
hsa-mir-196b	dbDEMC	hsa-mir-454	28795052
hsa-mir-99b	dbDEMC	hsa-mir-183	dbDEMC; HMDD v2.0
hsa-mir-142	25406066	hsa-mir-30e	unconfirmed
hsa-mir-203	dbDEMC; miR2Disease; HMDD v2.0	hsa-mir-342	dbDEMC; HMDD v2.0
hsa-mir-18b	dbDEMC;HMDD v2.0	hsa-mir-372	dbDEMC
hsa-mir-181a	dbDEMC; miR2Disease; HMDD v2.0	hsa-mir-95	dbDEMC
hsa-let-7c	dbDEMC;HMDD v2.0	hsa-mir-409	HMDD v2.0
hsa-mir-335	dbDEMC; miR2Disease; HMDD v2.0	hsa-mir-31	dbDEMC; miR2Disease; HMDD v2.0
hsa-mir-130a	dbDEMC	hsa-mir-192	dbDEMC
hsa-mir-199b	dbDEMC; HMDD v2.0	hsa-mir-96	dbDEMC; miR2Disease; HMDD v2.0
hsa-mir-29c	dbDEMC; miR2Disease; HMDD v2.0	hsa-mir-323	unconfirmed
hsa-mir-23b	dbDEMC;HMDD v2.0	hsa-mir-181d	dbDEMC; miR2Disease
hsa-mir-101	dbDEMC; miR2Disease; HMDD v2.0	hsa-mir-15b	dbDEMC
hsa-mir-224	dbDEMC;HMDD v2.0	hsa-mir-32	dbDEMC
hsa-mir-373	dbDEMC; miR2Disease; HMDD v2.0	hsa-mir-378	25120807

The first column records top 1–25 related miRNAs. The third column records the top 26–50 related miRNAs. The evidences for the associations were dbDEMC, miR2Disease and HMDD v2.0 or more recent experimental literatures with the corresponding PMIDs.
